# Quantifying the Role of Homophily in Human Cooperation Using Multiplex Evolutionary Game Theory

**DOI:** 10.1371/journal.pone.0140646

**Published:** 2015-10-23

**Authors:** Alessandro Di Stefano, Marialisa Scatà, Aurelio La Corte, Pietro Liò, Emanuele Catania, Ermanno Guardo, Salvatore Pagano

**Affiliations:** 1 Dipartimento di Ingegneria Elettrica, Elettronica e Informatica (DIEEI), Università degli Studi di Catania, Catania, Italy; 2 Computer Laboratory, University of Cambridge, Cambridge, United Kingdom; Kyushu University, JAPAN

## Abstract

Nature shows as human beings live and grow inside social structures. This assumption allows us to explain and explore how it may shape most of our behaviours and choices, and why we are not just blindly driven by instincts: our decisions are based on more complex cognitive reasons, based on our connectedness on different spaces. Thus, human cooperation emerges from this complex nature of social network. Our paper, focusing on the evolutionary dynamics, is intended to explore how and why it happens, and what kind of impact is caused by homophily among people. We investigate the evolution of human cooperation using evolutionary game theory on multiplex. Multiplexity, as an extra dimension of analysis, allows us to unveil the hidden dynamics and observe non-trivial patterns within a population across network layers. More importantly, we find a striking role of homophily, as the higher the homophily between individuals, the quicker is the convergence towards cooperation in the social dilemma. The simulation results, conducted both macroscopically and microscopically across the network layers in the multiplex, show quantitatively the role of homophily in human cooperation.

## Introduction

Charles Darwin observed how animals, from ants to people, interacting each other, are able to create social groups in which most of them work together for common good. By the way, it was in contrast with his idea of individual fitness surviving over the long term [1, 2]. This altruistic behaviour could be justified among kin in the natural selection mechanism. In [3], the authors explain that kin selection is conditioned by “kin recognition”, as an individual recognizes kin and behaves accordingly. Much research effort has been done in exploring this behaviour, but the understanding of how and why it may work out and evolve among people, linked by every type of relation, remains an open and major challenge. It raises the conundrum of cooperation, a widespread phenomenon in natural and social systems [4] but not fully-understood mainly due to its complexity. Cooperation produces a human conflict between the benefit of the single individual and that one of the population, such as risking one’s life to save a stranger. The reason why people do something for someone else, cooperating or helping, even though there is often a low probability for direct reciprocity or socially reward, is that actions are contagious [5].

Humans tend to cooperate building complex societies, as well as predators hunt in groups to catch more preys as possible [6]. More in general, cooperating means to contribute towards a common good at a cost to themselves, providing a benefit for others. Many models and mechanisms have been proposed to explain the emergence of cooperation, nevertheless, only by studying interactions inside population, we are able to explain the hidden patterns leading to cooperation [7, 8]. Cooperation may induce assortative interactions among individuals, transforming it into the most profitable strategy [9, 10].

Previous works on evolution of cooperation have identified some mechanisms, other than kin selection [11], related to interactions among individuals which can favour it, such as direct reciprocity, indirect reciprocity, spatial selection, and multilevel selection [12]. Direct reciprocity is related to a cost of cooperating in order to obtain a gain in the near future. Indirect reciprocity involves the dependence of an individual’s actions from the previous behaviours of the others. Spatial selection is linked with interactions and clusters of cooperators. Multilevel selection refers to competition existing between groups and between individuals. Rand and Nowak in [3] underline the importance to distinguish between interaction patterns that are mechanisms for the evolution of cooperation and behaviours that require an evolutionary explanation such as strong reciprocity, upstream reciprocity, and parochial altruism.

Therefore, how did the selfish process of natural selection give rise to cooperation? how might social interactions can give a boost to cooperative behaviour? And what may be the role of a linkage polarizer, such as *homophily*, in this evolutionary process? The evolution of cooperation among individuals is an unsolved puzzle: it has being observed since ancient times but, only in the recent years, a lot of research efforts have been done trying to understand and deepening the origin inside social networks. A vast literature on the evolution of cooperation on complex networks [13–16] highlights many aspects which offer insights on how cooperation can evolve and survive in different scenarios [17–21]. To study cooperation and its evolutionary dynamics, we need to understand the impact of the structure and the nature of social relationships among individuals. The study of network properties and dynamics is the result of a growing research interest in all the aspects related to social networks, from extracted data to emerging behaviours [22–25]. Therefore, both the structural and behavioural dimensions are fundamental to analyse what is the origin of the observed social dynamics within a population [26]. Social network analysis is intended to deepen the nature of nodes and ties [27], the actions and interactions between them and all the features and behaviours emerging from the combination of both aspects. These structural and behavioural dimensions allow to unveil the social contagion dynamics [28–31], showing how the influence runs through the ties connecting nodes, with regards to several phenomena at a population scale, such as diseases, smoking, happiness, etc. [32]. Then, network thinking is central in the analysis of contagion processes [33].

Social ties are crucial for collective action [34]. In [35] the authors have formalized the problem of collective action of large groups towards cooperative and uncooperative behaviours, considering how the role of a single actor or a group of people, community or coalition, could trigger a dynamic action within a population, which could represent a social contagion process. The most well-known theory of *critical mass* in the social sciences, is that by Granovetter [36], by considering people that have to make a binary choice, for instance, whether to join a protest or not, or whether to cooperate or not. In particular, it is argued that the large group problem can be solved by introducing the concept of critical mass, intended as the minimum number of initial contributors, whose efforts can produce a bandwagon effect, which has the power to involve the rest of population, for example, persuading the remaining members of the population towards the adoption of a specific behaviour [35]. Therefore, starting from a minority [37], the question is how many people should be involved in a collective action such that a single individual, interacting with them, becomes more likely to join the action? The answer is that each individual has his own threshold in terms of how many other people connected with him should join the action before he will do the same.

The actions of the nodes could be affected by a huge number of factors, among them one of the most important is the role played by homophily. The concept of homophily, that is the principle that similarity breeds connection, can explain how social connections are forged and severed over time [38]. In [39], the authors define homophily, in terms of information consumed and rumors spreading, as the tendency to interact with users and have similar consumption patterns. Homophily has been introduced and investigated in several works and across various domains [40–42] from friendship to information transfer. This concept generates some interesting behaviours observed in nature, shaping social relationships with a significant impact on information sharing, influence dynamics, and all the interactions people form and experience. Following this tendency to associate with others who are similar to them, we observe that a contact between similar people occurs at a higher rate that among dissimilar; in terms of social networks, this simply means that the attributes of vertices correlate across edges and it is known as assortative mixing. Among the various aspects of homophily, cognitive homophily is referred to the similarity in interests, beliefs, which can represent a reason towards a choice. People select each other because they share a similar representation of reality, strengthening some contacts rather than others. Nevertheless, other studies in the social sciences have pointed in the opposite direction, e.g. organizational ecologists have suggested that similarity can lead to competition for scarce resources [43], therefore competition among organizations using similar strategies, of similar size, and in geographical proximity with one another tends to be stronger than competition among dissimilar organizations [44, 45].

Furthermore, it is crucial to distinguish between homophily, social dependence and social influence. Homophily means that similar nodes are more likely to contact. Social dependence means that nodes exchange resources in order to satisfy their goals. Social influence means that nodes which interact become more similar.

Homophily or assortative mixing, however, is only a statement of pattern, and does not say much about the underlying mechanism. For example, if we observe a pattern of homophily in a social network, e.g., on political beliefs or socioeconomic status, we generally cannot distinguish between the edge forming as a result of the similar attributes, or the attributes becoming more similar as a result of the edge. The concept of homophily is important in the dynamics of collective action and critical mass mobilization [35]. Therefore, despite a lot of research efforts in studying the role of homophily in different fields [46], there is still much work to be done in studying its real effect on the evolution of social behaviours within a population.

**Fig 1:**
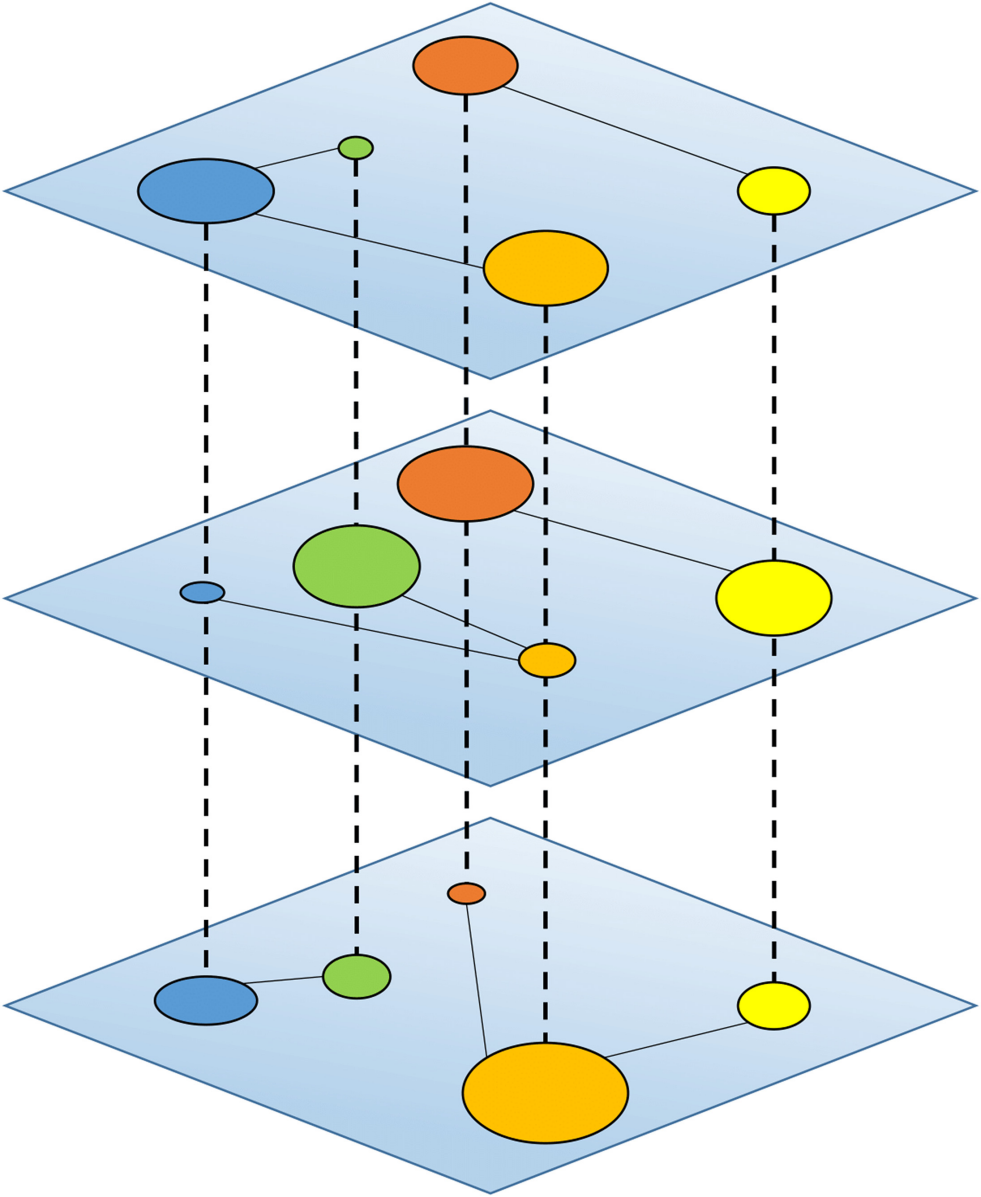
Schematic example of a multiplex network. The multiplex is made of N = 5 nodes embedded within M = 3 layers, each one containing 3 links. The size of nodes is proportional to centrality measure. The dashed lines represent interlayer connections, while the continuous lined represent the intra-layer connections.

From the other hand, homophily alone cannot explain why we connect or choose a strategy when interact with others, so it becomes essential considering the multiple types of relationships between nodes, known as multiplexity. In fact, the constituents of a huge variety of real-world complex systems, such as social networks, interact with each other following complicated patterns. Therefore multiplexity allows us to encompass these several interactions and relationships, exploring and unveiling how the different ties in the various layers can impact on the diffusion of social behaviours within a population. The presence of nodes in multiple layers of a system is the key to understand emergent phenomena, adding an extra dimension explaining what is the role not only of the intralayer interactions, as in a monoplex framework, but also of interlayer interactions for the emergence of these phenomena. Multiplex networks consist of multiple channels of connectivity, and they provide the more natural description for systems in which entities have a different set of neighbours in each layer [47] (see Fig 1).

In the social networks, these layers may correspond to different types of relationships: kin, co-workers, friends, etc. A fundamental aspect of describing multiplex networks is to quantify the interconnectivity between the different types of connections. In fact, interlayer connections can generate new structural and dynamical correlations between components of a system, and it is important to take them into account [48]. Multiplex networks are not just a particular case of interdependent networks [49], in fact, as in multiplex systems, many or even all of the nodes have a counterpart in each layer, so one can associate a vector of states to each node. In the multiplex case, the presence of nodes in multiple layers of a system also entails the possibility of self-interactions. This feature is absent in interdependent networks, which were generated as interconnected communities within a single, larger network [50–52]. In multiplex framework, being the same node at different layers has deep dynamical consequences and give rise to unexpected emergent phenomena [53].

To understand the evolution of social behaviours and, in particular, the emergence of human cooperation within a population in networks, it is important to have a mathematical framework to capture these underlying mechanisms. Fortunately, *Evolutionary Game Theory* (EGT) has provided a powerful framework to investigate cooperative behaviour in systems consisting of competitive individuals [54–56]. EGT allows to study interactions of multiple nodes in a population, and find out the hidden dynamics, shedding light on how and why some behaviours emerge following a specific pattern. Among the classical games, we consider the Prisoner’s Dilemma Game (PDG), that is one of the most common paradigms used to describe and study the problem of evolution [57, 58].

The investigation of evolutionary dynamics through EGT on multiplex networks allows unveiling and studying the existing social conflicts and dilemmas among the interests of the single nodes and groups, their counterparts in various layers, not neglecting what is captured from homophily, the patterns of similarity and dissimilarity [6, 59].

In this work, we targeted at investigating the evolutionary dynamics of human cooperation dilemma considering the multiplexity of interactions between nodes.

To explore the nature of human cooperation, we take into account a Critical Mass [34, 35], able to pop up a new behaviour and trigger a collective action within a population. To analyse the contagiousness of the action [28], we investigate the social connectedness, using a multiplex evolutionary game theory framework [59] and bringing out the real reason why similarity breeds connection [38]. Therefore, we focus on both the role of homophily and multiplexity [60, 61], stressing also the importance of the coupling between layers using the communicability function inside the multiplex network [62]. Taking into account all these aspects, we propose a novel analytical model and simulate the evolution of human cooperation using evolutionary game theory.

Our work, coherently with [6, 59], is intended to analyse the problem of the emergence of cooperation in multiplex networks using EGT. Our findings highlight the key role played by homophily and multiplexity in the evolution of cooperation. In fact, despite the apparently constrained nature of homophily in reducing the boundaries of connectedness, homophily allows to observe a new nature of the interaction patterns people experience, looking at these patterns through multiplexity.

## Materials and Methods

### Critical Mass, Centrality and Homophily in Multiplex Network

Critical mass is defined as the minimum coalition *min*(*n*), such that if actors organize into coalitions of size *n*, at least *n* people will prefer mutual cooperation to unilateral defection, and it is calculated as follows [35]:

$$min\left(n\right)\;s.t.\{\sum_{i=1}^{N}H\left(R_{i}-T_{i}\right)\}\geq n$$ (1)

where *n* is the overall population and *min*(*n*) is the minimum coalition size. The latter depends on the Heaviside function of the difference between Reward and Temptation payoffs, *R*_*i*_ and *T*_*i*_ respectively, evaluated considering different types of games [35].

In this work we extend the concept of Critical Mass introducing a social network approach, considering a scale-free network [63], and taking into account centrality and homophily measures in a multiplex structure. Furthermore, we aim not only to evaluate the minimum coalition size, but also to define a new kind of “Critical Mass” (*CM*), as the minimum information enclosed in one or more nodes’ configurations able to trigger a diffusion process of a behaviour within a population. This represents the role of *CM* for investigating the human cooperation. The idea is to observe and track the diffusion of behaviours between connected nodes using a multiplex approach.

**Fig 2:**
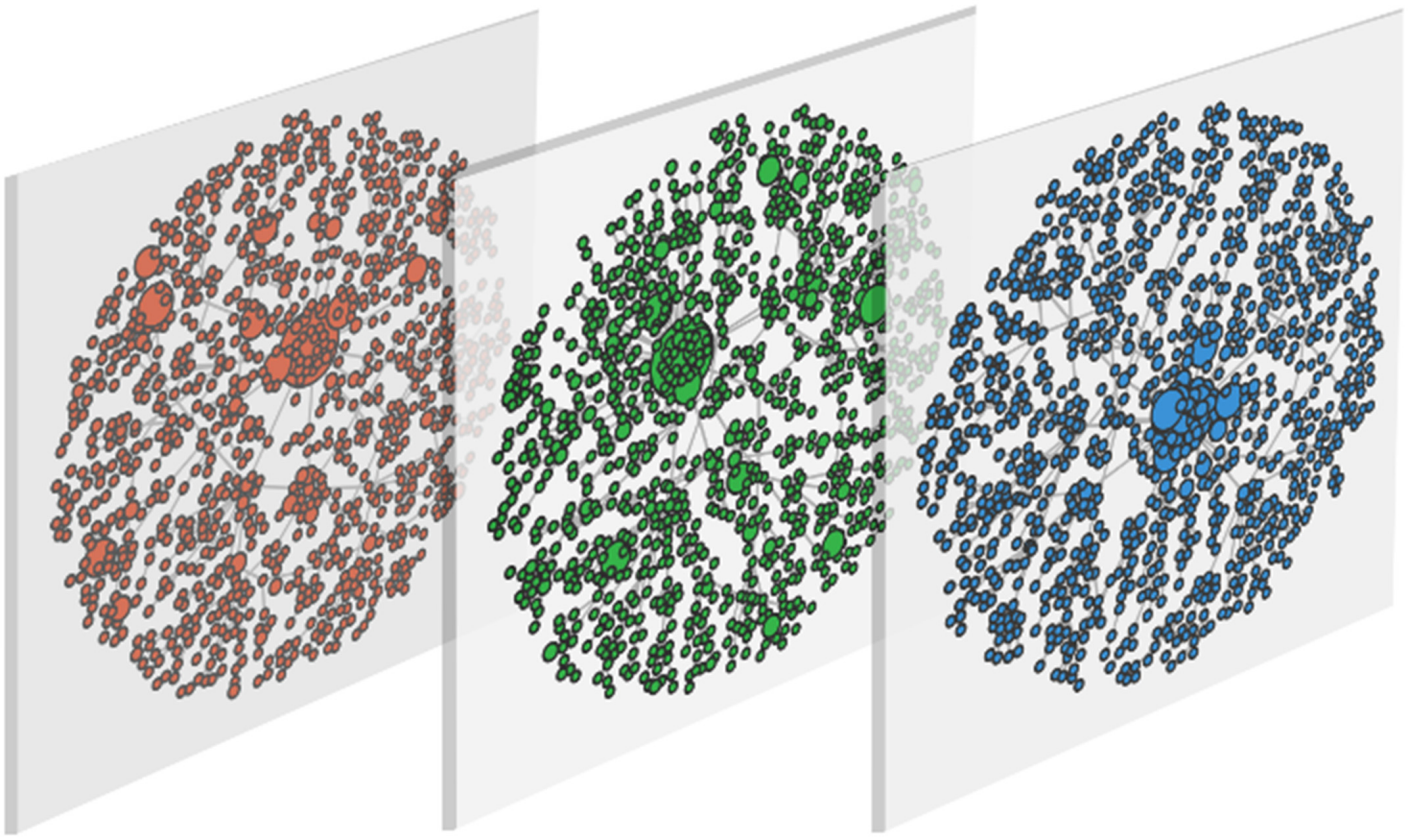
Centrality distribution in the multiplex network. The multiplex is made of *N* = 1000 nodes embedded within *M* = 3 layers, each one modelled by a different scale-free network. The size of nodes is proportional to centrality measure.

We take into account a scale-free network, thus *CM* is transformed into a set of nodes that depends on the network structure taking into account centrality. In particular, we choose the eigenvector-like centrality measure, which is defined in [64]. The eigenvector-like centrality allows to include the concept of influence in our analysis; starting from the spectral properties of the adjacency matrix, considers not only the number of links of each node, but also the quality of such connections. Central nodes are the most influential nodes which can condition the behaviours of their neighbouring nodes. In Fig 2 we show the centrality measures and its distribution in the multiplex network.

The analysis of *CM* is then further extended considering a multiplex structure M, taking a different eigenvector-like centrality measure in each layer *α*, in order to consider different degrees of importance (or influence) in different layers of the network, and to include this information in the definition of a matrix of mutual influence between layers. Thus, to calculate the centrality of a node within a specific layer, one must take into account all the other layers, as it may depends not only on the neighbours that are linked to *x* ^*α*^ within that layer, but also on all other neighbours of *x* ^*β*^ that belong to the other layers. In other words, one needs to consider the situation where the influence amongst layers is heterogeneous. To this aim, one can introduce an influence matrix *W*, defined as a non-negative matrix, such that *W* ^*αβ*^ measures the influence on a layer *α* given by the layer *β*. Given a multiplex network M and an influence matrix *W* = (*w* ^*αβ*^), we define the global heterogeneous eigenvector-like centrality of M as in [65].

For each layer *α*, we introduce the adjacency matrix, denoted by $A^{\alpha }\in R^{N\times N}$, where each element is:

$$a_{xy}^{\alpha }=a_{yx}^{\alpha }=\{\begin{array}{cc} 1, & \text{if}\;x\;\text{and}\;y\;\text{are}\;\text{connected}\\ 0, & \text{otherwise} \end{array},\;\text{for}\;1\leq \alpha \leq M$$ (2)

Now we extend the homophily measure considering a multiplex structure. In each layer *α*, we define an Homophily matrix *H* ^*α*^, where each element $h_{xy}^{\alpha }$ represents the homophily measure between two nodes *x* and *y* in the layer *α*, calculated as:

$$h_{xy}^{\alpha }=\frac{1}{1+\delta_{xy}^{\alpha }}$$

where *δ*_*xy*_ measures the homophily difference between two nodes *x* and *y*. Then, the Homophily matrix is defined as follows:

$$H^{\alpha }=[\begin{array}{ccc} 1 & \cdots & \frac{1}{1+\delta_{1,N}^{\alpha }}\\ \vdots & \ddots & \vdots \\ \frac{1}{1+\delta_{N,1}^{\alpha }} & \cdots & 1 \end{array}]\in R^{N\times N}$$ (3)

For each layer *α*, we define the matrix *Z* ^*α*^, as the Hadamard product between the homophily matrix *H* ^*α*^ and the adjacency matrix *A* ^*α*^, as follows:

$$Z^{\alpha }=H^{\alpha }\circ A^{\alpha }=[\begin{array}{ccc} 0 & \cdots & \frac{a_{1,N}^{\alpha }}{1+\delta_{1,N}^{\alpha }}\\ \vdots & \ddots & \vdots \\ \frac{a_{N,1}^{\alpha }}{1+\delta_{N,1}^{\alpha }} & \cdots & 0 \end{array}]\in R^{N\times N}$$ (4)

where each element is given by:

$$z_{xy}^{\alpha }=\frac{a_{xy}^{\alpha }}{1+\delta_{xy}^{\alpha }}$$

Note that *Z* ^*α*^ degenerates in the adjacency matrix *A* ^*α*^ if, for each pair of nodes, we have $\delta_{xy}^{\alpha }=0$, that is a network with no homophily difference between nodes. In order to obtain an overall measure that includes both the concepts of centrality and homophily in the multiplex structure, in a first step we need to evaluate the global heterogeneous eigenvector-like centrality and homophily of the multiplex M, defined as a positive and normalized eigenvector $o^{⊗}\in R^{NM}$ (if it exists) of the matrix:

$$Z^{⊗}=[\begin{array}{cccc} w^{11}\;{\left(Z^{1}\right)}^{T} & w^{12}\;{\left(Z^{2}\right)}^{T} & \cdots & w^{1M}\;{\left(Z^{M}\right)}^{T}\\ w^{21}\;{\left(Z^{1}\right)}^{T} & w^{22}\;{\left(Z^{2}\right)}^{T} & \cdots & w^{2M}\;{\left(Z^{M}\right)}^{T}\\ \vdots & \vdots & \ddots & \vdots \\ w^{M1}\;{\left(Z^{1}\right)}^{T} & w^{M2}\;{\left(Z^{2}\right)}^{T} & \cdots & w^{MM}\;{\left(Z^{M}\right)}^{T} \end{array}]\in \;R^{\left(NM\right)\times (NM)}$$ (5)

where *Z* ^⊗^ is the Khatri–Rao product of the matrices:

$$W=[\begin{array}{cccc} w^{11} & w^{12} & \cdots & w^{1M}\\ w^{21} & w^{22} & \cdots & w^{2M}\\ \vdots & \vdots & \ddots & \vdots \\ w^{M1} & w^{M2} & \cdots & w^{MM} \end{array}]$$ (6)

and

$$Z^{T}=[\begin{array}{cccc} {\left(Z^{1}\right)}^{T} & {\left(Z^{2}\right)}^{T} & \cdots & {\left(Z^{M}\right)}^{T} \end{array}]$$ (7)

Note that we consider a symmetric homophily measure between two different nodes, that is *δ*_*xy*_ = *δ*_*yx*_. In other words, we consider realistically that, in terms of similarity, two connected nodes present a symmetric measure, so that: (*Z* ^*α*^)^*T*^ = *Z* ^*α*^.

Introducing the following notation:

$$o^{⊗}=[\begin{array}{c} o^{1⊗}\\ o^{2⊗}\\ \vdots \\ o^{M⊗} \end{array}]\in R^{NM}$$ (8)

where $o^{1⊗},\;o^{2⊗},\;\cdots ,\;o^{\alpha ⊗},\;\cdots ,\;o^{M⊗}\in R^{N}$, we can define the global heterogeneous eigenvector-like matrix *O* ^⊗^ of M, as follows:

$$O^{⊗}=[\begin{array}{cccc} o^{1⊗} & o^{2⊗} & \cdots & o^{M⊗} \end{array}]\in R^{N\times M}$$ (9)

Once defined Eq (9), in a second step, for each node *x*, we define an overall measure of its centrality and homophily, denoted by *λ*_*x*_, in the multiplex network M. Λ is a column vector of size *N*, which includes all the measures *λ*_*x*_. It allows to quantify the overall weight, in terms of centrality and homophily, of each node in the multiplex M, as follows:

$$\Lambda =[\begin{array}{c} \lambda_{1}\\ \lambda_{2}\\ \vdots \\ \lambda_{N} \end{array}]=[\begin{array}{c} \sum_{i=1}^{M}{\left(o^{i⊗}\right)}_{1}\\ \sum_{i=1}^{M}{\left(o^{i⊗}\right)}_{2}\\ \vdots \\ \sum_{i=1}^{M}{\left(o^{i⊗}\right)}_{N} \end{array}]\in R^{N}$$ (10)

Note that:

$$\sum_{x=1}^{N}\lambda_{x}=1$$

Now we want to define the *CM* in the multiplex structure both in a quantitative and qualitatively way. To this purpose, on one hand, we evaluate the minimum coalition size $\bar{n}$ and, on the other hand, we also identify the nodes which maximize the diffusion process of a behaviour within a population of size *N*.

First we consider the multiplex as a single layer of *N* × *M* nodes, and we calculate the *CM* size $\bar{n}$, as follows:

$$min(\bar{n})\;s.t.\;(\sum_{i=1}^{NM}H\;(R_{i}-T_{i}))\geq \bar{n}$$ (11)

We identify a node as “critical” when it triggers a certain behaviour in all the layers in which it is involved. Therefore, the *CM* results in a set of “critical” nodes able to give a boost to a certain behaviour in a more effective and faster way, due to its high centrality and homophily weight. In fact, more a node is central in the network structure and more it is similar to the other nodes in the different layers of the multiplex structure, more it becomes relevant in triggering a behaviour.

Therefore, assumed that in the multiplex network a “critical” node adopts the same behaviour in all the layers, starting from Eq (11) and considering a multiplex network, the *CM* size $\bar{\bar{n}}$ of M is given by:

$$\bar{\bar{n}}=\frac{\bar{n}}{M}\leq n$$ (12)

as we can exclude the replicas of a node that belongs to the set of *CM* nodes. In other words, $\bar{n}$ is the *CM* size of the “aggregate layer”, obtained considering in a single layer all the connections in the different layers and the nodes in the multiplex (including its counterparts in the different layers of the multiplex). Therefore, the definition of $\bar{\bar{n}}$ allows us to leave out the critical nodes’ counterparts from the *CM* size.

We define the Critical Mass density, denoted by *τ*_*CM*_, as follows:

$$\tau_{CM}=\frac{\bar{\bar{n}}}{N}$$ (13)

**Fig 3:**
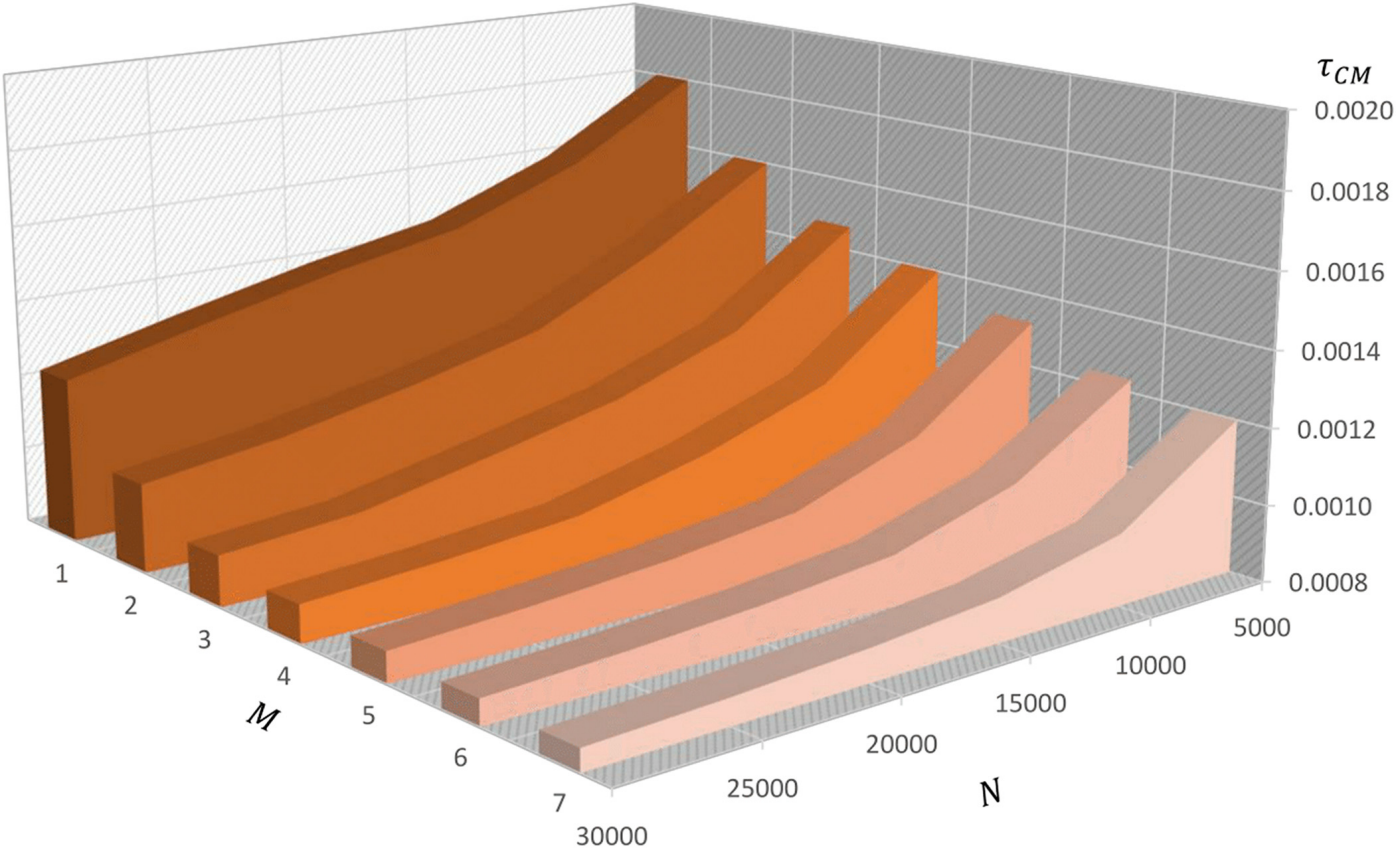
Critical mass density. The Critical Mass Density as a function of the population’s size *N* and the number of layers *M*.

In Fig 3 we show the *CM* density *τ*_*CM*_ according to the population of size *N* and the number of layers *M*. The plots are generated considering a population *N* ranging from 5000 nodes to 30000 nodes, and a number of layers *M* ranging from one layer to seven layers.

The boundaries of the variation ranges related to the variables *N* and *M* are due to the convergence process of the *τ*_*CM*_ to a limit value denoted by ${\bar{\tau }}_{CM}$. In fact, although increasing the size of the “aggregate layer”, *τ*_*CM*_ maintains nearly the same value:

$$\lim_{N\times M\to +\infty }\tau_{CM}={\bar{\tau }}_{CM}$$ (14)

The plot highlights how the *τ*_*CM*_ decreases with the population and the number of layers in the considered variation intervals. Thus, these findings shed light on the significance of the inequality.

To identify qualitatively the set of *CM* nodes, we take into account the set *R*, representing all the permutations *S*_*k*_ of the $\bar{\bar{n}}$ nodes in the population *N*, so that we overall have $N!/\bar{\bar{n}}!$ permutations.

The set *R* is defined as follows:

$$R=\{S_{1},S_{2},...S_{N!/\bar{\bar{n}}!}\}$$ (15)

For each subset *S*_*k*_ of *R*, we can define an overall centrality and homophily measure, as follows:

$$\Lambda_{S_{k}}=\sum_{x\in S_{k}}\lambda_{x}$$ (16)

The *CM* in the multiplex structure M, indicated with $\bar{S}$, is the subset which maximizes the overall centrality and homophily measure, that is $\Lambda_{\bar{S}}$, as follows:

$$\bar{S}\in R\;s.t.\;\Lambda_{\bar{S}}=\underset{S\in R}{arg max}\{\sum_{x\in S}\lambda_{x}\}$$ (17)

Note that it may be more than one subset that satisfy the Eq 17. Furthermore, from a computational point of view, calculating $\bar{S}$ is simple, since we have only to consider the $\bar{\bar{n}}$ nodes with the higher values of *λ*_*x*_.

### Communicability in Multiplex

In our work, we want to stress the importance of the coupling between layers in exploring the evolution of behaviours in the multiplex structure. To this aim we exploit the communicability function defined in [62], which quantifies the number of possible routes that two nodes have to communicate with each other.

Therefore, considering a multiplex formed by *M* layers, denoted by *L*_1_, *L*_2_, …, *L*_*M*_, and their respective matrices *Z*_1_, *Z*_2_, …, *Z*_*M*_, representing the Hadamard product between the homophily matrices and the adjacency matrices of the multiplex M, its matrix is then given by M = *Z*_*L*_ + *C*_*LL*_, where *Z*_*L*_ is:

$$Z_{L}={⊕}_{a=1}^{M}Z_{\alpha }$$ (18)

and *C*_*LL*_ is a matrix describing the interlayer interaction, defined as follows:

$$C_{LL}=[\begin{array}{cccc} 0 & C_{12} & ... & C_{1M}\\ C_{21} & 0 & ... & C_{2M}\\ \vdots & \vdots & \ddots & \vdots \\ C_{M1} & C_{M2} & ... & 0 \end{array}]\in R^{NM\times NM}$$ (19)

where each element $C_{\alpha \beta }\in R^{N\times N}$ represents the interaction of layer *α* with layer *β*. Here it is assumed that: *C*_*αβ*_ = *C*_*βα*_ = *C* = *ω*_*αβ*_ *I* = *ω*_*βα*_ *I*, for all layers *α* and *β*, as we consider a symmetric interaction between layers. *ω* is the parameter describing the strength of the interlayer interaction, and $I\in R^{N\times N}$ is the corresponding identity matrix. So we can now explain the multiplex matrix as follow:

$$M=[\begin{array}{cccc} Z_{1} & \omega_{12}I & ... & \omega_{1M}I\\ \omega_{21}I & Z_{2} & ... & \omega_{2M}I\\ \vdots & \vdots & \ddots & \vdots \\ \omega_{M1}I & \omega_{M2}I & ... & Z_{M} \end{array}]\in R^{NM\times NM}$$ (20)

Since we are interested in accounting for all the walks between any pair of nodes in the multiplex, we consider the number of walks of length *k* between two generic nodes *x* and *y* in the multiplex, which is given by the *α*, *β*-entry of the *K*-th power of the adjacency matrix of the network. Consequently, the walks of *k* length in the multiplex are given by the different entries of M^*K*^. As underlined in [62], the walks can include hops of two different kinds, e.g., intra-layer and interlayer hops, and we are interested in giving more weight to the shortest walks than to the longer ones. The communicability between two nodes *x* and *y* in the multiplex is given by a weighted sum of all walks from *x* to *y* as follows:

$$G_{xy}=I+M+\frac{M^{2}}{2!}+...=\sum_{k=0}^{\infty }\frac{M^{k}}{k!}={\left[exp(Z_{L}+C_{LL})\right]}_{xy}$$ (21)

Now we introduce the communicability matrix *G*, where each element $G_{\alpha \beta }\in R^{N\times N}$ is the matrix representing the communicability between every pair of nodes belonging to two different layers *α* and *β*, of the multiplex M. It is defined as follows:

$$G=exp\left(Z_{L}+C_{LL}\right)=[\begin{array}{cccc} G_{11} & G_{12} & ... & G_{1M}\\ G_{21} & G_{22} & ... & G_{2M}\\ \vdots & \vdots & \ddots & \vdots \\ G_{M1} & G_{M2} & ... & G_{MM} \end{array}]\in R^{NM\times NM}$$ (22)

In particular, [*G*_*αβ*_]_*xy*_ represents the communicability between the node *x* in the layer *α* and the node *y* in the layer *β*.

### Evolutionary Dynamics

We use the Prisoner’s Dilemma game (PDG) as a general metaphor for studying the evolution of cooperation. In this classical social dilemma, two players simultaneously decide whether to cooperate (*C*) or to defect (*D*): cooperation results in a benefit *b* to the opposing player, but incurs a cost *c* to the cooperator (where *b* > *c* > 0); defection has no costs or benefits. In both cases, it is best to defect for rational individuals in a single round of the PDG, regardless of the opponent strategy. However, mutual cooperation leads to a higher payoff than mutual defection, but cooperation is irrational. The social dilemma is thus established, since mutual cooperation yields both an individual and total benefit higher than that of mutual defection. The payoff matrix of the PDG is illustrated in Table 1:

**Table 1:** Payoff Matrices of the Prisoner’s Dilemma Game.

	Cooperate	Defect
Payoff to Cooperation	*b* − *c*	−*c*
Payoff to Defection	*b*	0

In evolutionary settings, payoffs determine reproductive fitness, and it follows that *D* is the *Evolutionarily Stable Strategy* (ESS). This can be formalized using replicator dynamics [56], which admits pure defection as the only stable equilibrium. The *PD* game is in fact the most stringent cooperative dilemma where, for cooperation to arise, a mechanism for the evolution of cooperation is needed [66]. The pairwise nature of the game is translated to a population scale by making the nodes playing with each other, and accumulating the payoff obtained from each interaction. After each round of the game, the strategies of the nodes are updated so that those nodes with less payoff are tempted to imitate the strategy of those fittest individuals. We focus on memory-one game since in [67] the authors have proved that, giving only a finite memory of previous play, the payoff obtained is exactly the same as if we would consider a player with a longer memory. In unstructured populations, in which players are well-mixed, evolutionary dynamics leads all the individuals to defection [65]. However, the existence of a network of interactions, so that each node can only play with those directly connected to it, the population can sometimes promote the emergence of cooperation. This mechanism promoting cooperation, known as network reciprocity [68, 69], was observed to be substantially enhanced when the network substrate is a scale-free network [13, 66], a real-world network, with a power law dependence of the degree distribution *P*(*k*) ∼ *k* ^*γ*^, with the exponent *γ* typically satisfying 2 < *γ* < 3. For this reason, we decide to adopt a scale-free as network substrate [63].

We simulate the evolutionary process in accordance with the standard Monte Carlo simulation procedure, composed of elementary steps; including the distribution of competing strategies, which is an elementary step entails randomly selecting a player and one of its neighbours, calculating the payoffs of both players, and finally attempting a strategy adoption. First, a randomly selected player *x* acquires its payoff *P*_*x*_ by playing the game with all its neighbours on the layer *α*. Next, player *x* randomly chooses one neighbour *y* on the layer *β*, who then also acquires its payoff *P*_*y*_ on the layer *β* in the same way as previously did player *x*. Lastly, player *x* adopts the strategy *S*_*y*_ from player *y* with a probability determined by the Fermi function [70]:

$$W\left(S_{y}\to S_{x}\right)=\eta_{x}\frac{1}{1+exp\;[\frac{P_{x}-P_{y}}{\delta_{xy}K}]}$$ (23)

One player *x* on the layer *α* of the multiplex M adopts the strategy *S*_*y*_ of another node playing on the layer *β*, taking into account the payoff difference, the homophily measure *δ*_*xy*_ and a communicability measure *η*_*x*_ in the multiplex network. We take into account a degree of uncertainty in the decision making process given by the factor *K*. In fact, the temperature *K* represents a noise level (or selection intensity) and quantifies the uncertainty related to the strategy adoption process; it can vary in the range [0, +∞]. The selected value of *K* is a traditional and frequently employed choice that does not qualitatively affect the evolutionary outcomes, as shown in many preceding works and reviewed comprehensively in [71]. In the *K* → 0 limit, the adoption of a successful strategy is deterministic, while in the *K* → +∞ limit, the strategy learning is blind. The factor *δ*_*xy*_, related to the homophily measure, means that the more the players have a high value, the more one player tends to imitate the strategy of the other one at each round. *δ*_*xy*_ can vary in the range [0, +∞]; in particular for *δ*_*xy*_ → 0, the two nodes present the highest homophily value, while in the *δ*_*xy*_ → +∞ limit, there is no homophily. Furthermore, we introduce the scaling factor *η*_*x*_ of player *x* which depends on the strategies of related players from the other layers, and it is the key quantity that takes into account the communicability function between layers [15]. If we consider only the strategy of the counterpart *x*′ on another layer *β*, we are in the simplest case and we can assume that *η*_*x*_ is minimal if *S*_*x*_ = *S*_*x*′_, otherwise it assumes the maximal value. To avoid frozen states the scaling factor ranges in the interval [0.1, 1], assuming *η*_*x*_*min*__ = 0.1 as the minimal scaling factor and *η*_*x*_*max*__ = 1 as the maximal value.

In our definition, we consider a more general case where not only the counterpart node *x*′ but also its neighbours on the other layer *β* determine *η*_*x*_; in other words, the counterpart and its neighbours can influence the strategy adoption due to the communicability, that includes the interlayer interaction and the number of possible walks from node *x* to *y*, where *y* are all the neighbouring nodes connected with the counterpart node *x*′ on the layer *β* of the node *x* on the layer *α*.

The scaling factor *η*_*x*_ changes linearly between *η*_*x*_*min*__ and *η*_*x*_*max*__ in accordance with:

$$\eta_{x}=1-\left(\eta_{x_{max}}-\eta_{x_{min}}\right)\frac{\sum_{y\in \beta ,S_{y}=S_{x}}{\left[G_{\alpha \beta }\right]}_{xy}}{\sum_{y\in \beta }{\left[G_{\alpha \beta }\right]}_{xy}}$$ (24)

where the numerator is the sum of the communicability functions calculated between the node *x* on the layer *α* and all the neighbouring nodes *y* belonging to the layer *β*, adopting the same strategy as player *x*. While the denominator represents the sum of the communicability functions calculated between the node *x* on the layer *α* and all the neighbouring nodes *y* belonging to the layer *β*. Therefore, the ratio quantifies the influence, in terms of communicability, on the strategy adoption of the player *x* on the layer *α*, due to the strategies adopted by the counterpart node and its neighbours on the layer *β*. In particular, more are players on the layer *β* with a high communicability with the node *x* adopting the same strategy as player *x*, more likely *x* will adopt the same strategy in the next round. On the other hand, if there are nodes on the layer *β* with a high communicability, but adopting a different strategy, the player *x* will be most likely pushed to change its strategy. Thus, this ratio depends on the communicability function and it may result in a bias regarding the strategy adoption of the player *x* in the next round of the game. Each Monte Carlo step gives a chance for every player to change its strategy once on average.

## Results and Discussion

**Fig 4:**
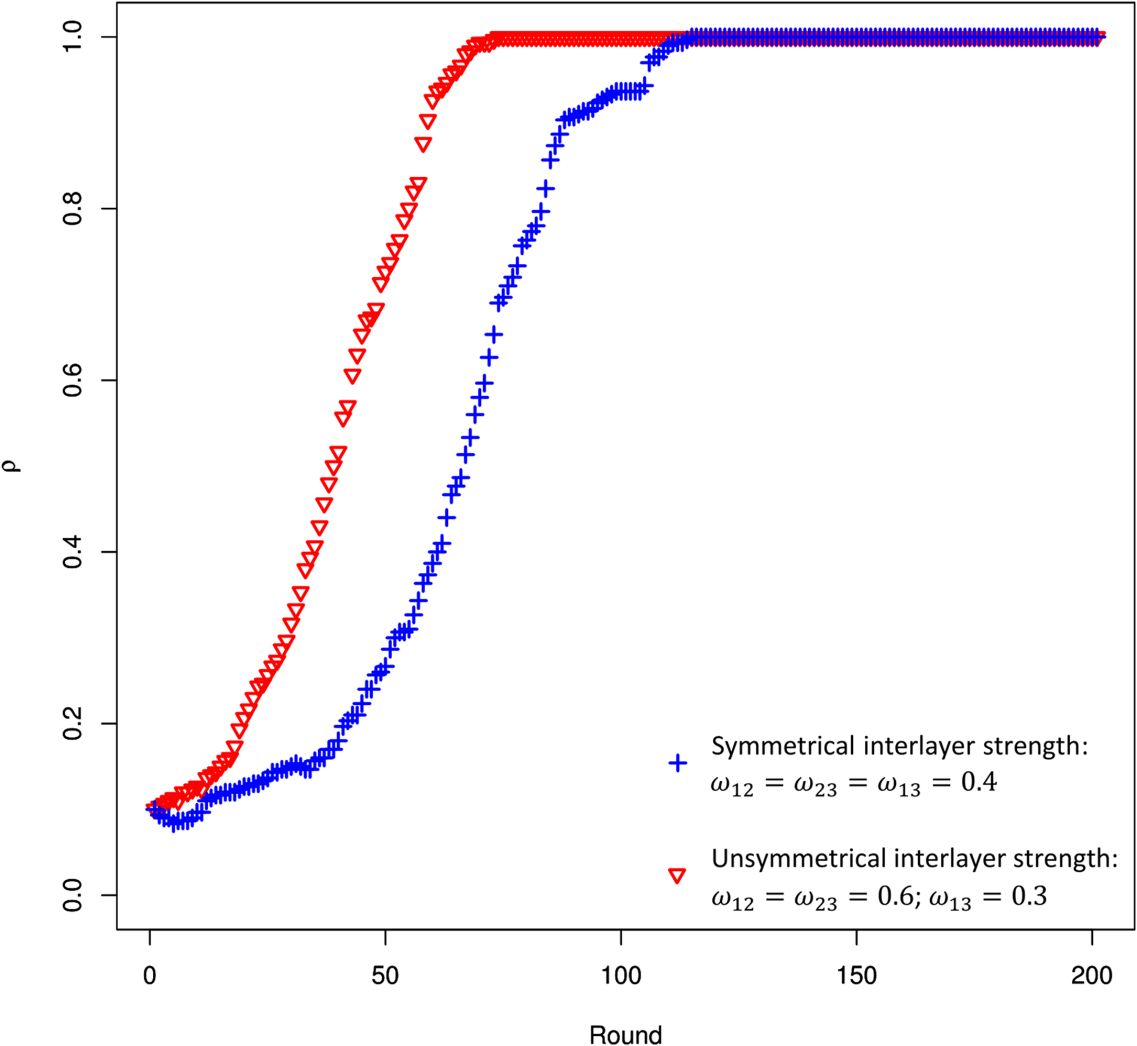
Evolution of cooperation considering different interlayer interaction strength. The evolution of cooperation against the round as a function of interlayer interaction strength. The ‘blue’ plot represents the case of constant interlayer strength: *ω*_*αβ*_ = 0.4. The ‘red’ plot represents the case of variable interlayer strength (one dominant layer): *ω*_*αβ*_ = 0.3 between layers 1 and 3; *ω*_*αβ*_ = 0.6 between the layer 2 and the other layers of the multiplex. We show the evolution of cooperation until 200 rounds as, in correspondence of that value, the convergence has already been reached. It can be observed that the emergence of cooperation is quicker considering a variable interlayer strength (one dominant layer), than the constant case. The dominant layer acts as a behaviour’s polarizer of the nodes in the other layers.

The simulations have been conducted choosing a scale-free network with *N* = 1000 nodes. We take into account different values of homophily randomly chosen following a normal distribution around a mean value, with standard deviation *σ*. Furthermore, we have considered two different values of interlayer interaction strength *ω*_*αβ*_; in particular, *ω*_*αβ*_ = 0.3 indicates a low interlayer interaction strength between the layers 1 and 3, while *ω*_*αβ*_ = 0.6 represents a high interlayer interaction strength between the layers 2 and the others. The reasons behind our choice of the interlayer interaction strength between layers are explained in Fig 4.

**Fig 5:**
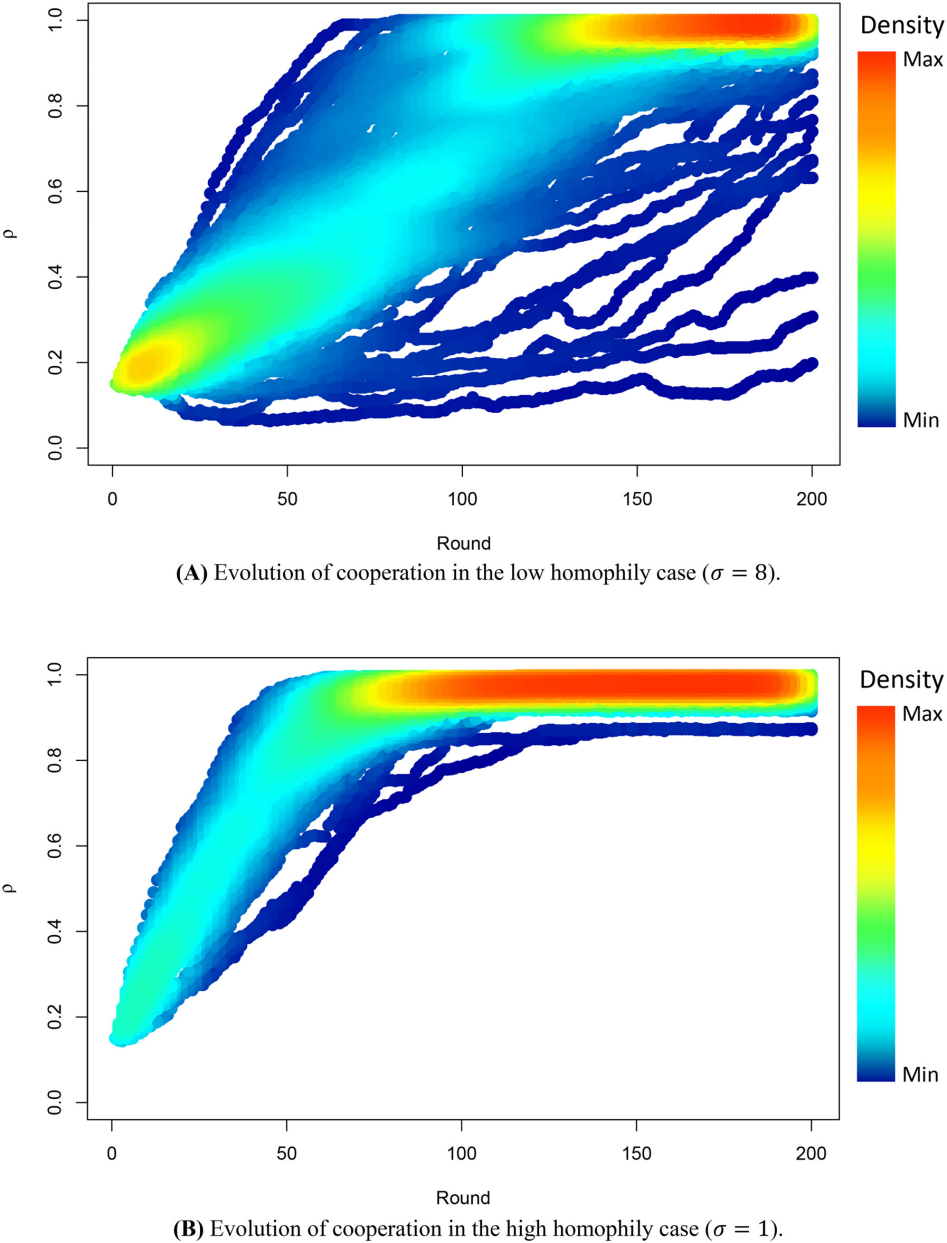
Emergence of cooperation over time. The figure illustrates the fraction of cooperative nodes against the rounds or time steps: low homophily (**A**) and high homophily (**B**). The figure shows the evolutionary dynamics of the PD game played between the interacting nodes in a multiplex network with *M* = 3 layers. In both cases *N* = 1000 nodes. The results are obtained choosing a fixed number of simulations and the colour corresponds to the density: ‘red’ indicates the highest density (that is the maximum number of overlapping points), while ‘blue’ means the lowest density. As can be observed, increasing the homophily value of the multiplex network M, we note a faster emergence of cooperation.

Fig 5 shows the fraction (or density) of cooperative nodes against the rounds or time steps. *ρ* varies in the range [0, 1], where 0 corresponds to the global defection, while 1 means a global cooperation of population. We have simulated the evolutionary dynamics for a fixed number of simulations, and the colour corresponds to the population’s density, so ‘red’ indicates the highest density, while ‘blue’ means the lowest density. In Fig 5, the PD game is played between the interacting nodes in a multiplex network with *M* = 3 layers. We have considered two different values of *σ*, where *σ* = 8 means a low homophily value (Fig 5A), while *σ* = 1 means a higher homophily value (Fig 5B), fixed a *CM* value. We show the evolution of cooperation until 200 rounds as, in correspondence of that value, the convergence has already been reached.

**Fig 6:**
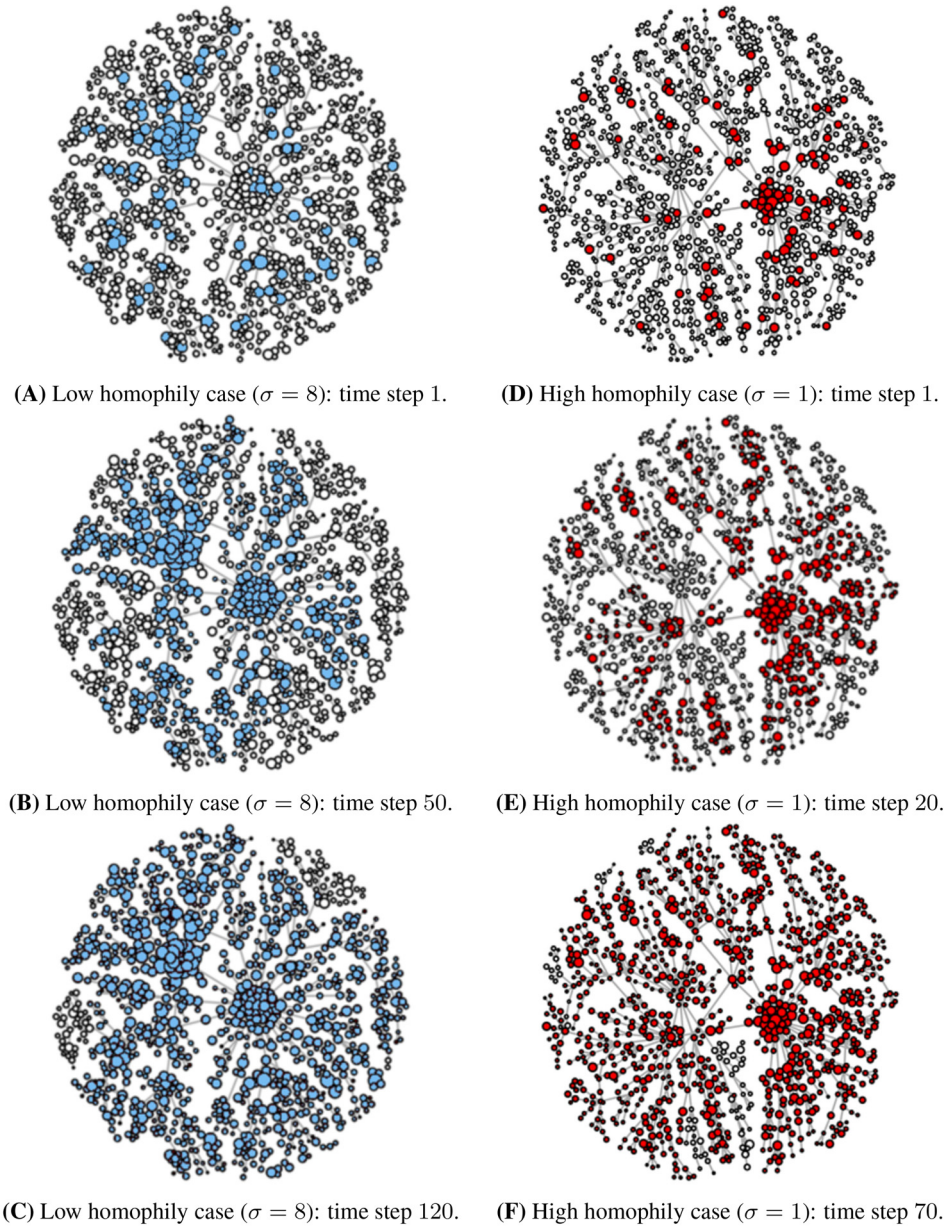
Temporal evolution of cooperation. The figure highlights the microscopic emergence of cooperation in the evolutionary process. The formation of cooperative groups in the network and also the group size depend on the homophily value. Figs **A**, **B**, **C**—in the low homophily case (*σ* = 8), the defective behaviour tends to persist more in the population, not favouring the formation of cooperative groups and globally slowing the emergence of cooperation. Yet, the group size will be smaller in this case of low homophily. Figs **D**, **E**, **F**—in the high homophily case (*σ* = 1), the convergence towards cooperation becomes quicker, and there is a natural formation of larger cooperative groups than in low homophily case. Analysing the corresponding figures of the evolution, we see clearly this difference, both in speed and size, in the formation of cooperative groups.

This macroscopic evolution highlights how the higher is the homophily value, more quickly nodes converge to cooperation and the density of cooperative nodes tends to the maximum value. In other words, increasing the homophily value of the multiplex network M, we note a faster emergence of cooperation. Instead, considering lower homophily values, we find a lower density of cooperative nodes, that means a slower convergence to cooperation. The results are coherent with our theoretical expectations: in fact the more the homophily, the more the nodes tend to choose the same cooperative strategy, solving the social dilemma towards the most profitable strategy with the highest payoff for the evolutionary fitness of population. The switching from the pure rational strategy to the most profitable one is due to the interaction between nodes through the different layers of the multiplex network, creating a sort of ‘learning process’ driven by homophily, which acts as a catalyst towards cooperation. In Fig 6 we illustrate the microscopic evolution of cooperation, considering both the cases, respectively with low homophily (*σ* = 8) and high homophily value (*σ* = 1). We have simulated the evolution of cooperation in the multiplex network, showing the evolutionary dynamics of one of the layers, as the evolution in one layer is representative of the overall one in all the layers of the multiplex.

During the steps of evolutionary dynamics, the nodes becomes coloured when they cooperate, otherwise they are ‘white’. In particular, we coloured in ‘blue’ the cooperative nodes in the case of low homophily *σ* = 8, while we indicated with ‘red’ the case of high homophily *σ* = 1. The size of nodes are log-proportional to the values of Λ (see Eq 10), so it depends on both the centrality and homophily measures of the multiplex network (see Materials and Methods). As in Fig 5, the Fig 6 highlights the different speed in the emergence of cooperation of the evolutionary process. The formation of cooperative groups in the different parts of the network and also the group size depend on the homophily value. When we consider a low homophily value, nodes tend not to interact with the others in the multiplex network, then the defective behaviour tends to persist more in the population, not favouring the formation of cooperative groups and globally slowing the emergence of cooperation. As a consequence, the group size will be small in this case of low homophily (see Fig 6A, 6B and 6C). Instead, when we consider a high homophily value, nodes are pushed to interact with each other, so the convergence towards cooperation becomes quicker, and there is a natural formation of larger cooperative groups than in low homophily case (see Fig 6D, 6E and 6F). Analysing concurrently the corresponding figures of microscopic evolution, we see clearly this difference, both in speed and size, in the formation of cooperative groups. In both cases of respectively low and high homophily, we illustrate the evolutionary process until the convergence has already been reached.

To sum up, starting from [6, 59], we analysed the emergence of cooperation in multiplex networks. To this aim, we defined a novel analytical model able to analyse the problem of human cooperation in multiplex networks using evolutionary game theory, exploring the role played by multiplexity and homophily in the evolution of cooperation. Therefore, first we have introduced the critical mass in a multiplex network, proposing also the selection criterion to detect nodes to trigger the evolution. To capture the effect of multiplexity and stress the importance of the coupling between the network layers, we have exploited the communicability function defined in [62]. We observed how the emergence of cooperation is quicker considering a variable interlayer strength in the different layers, with one dominant layer, than the constant case with the same interlayer strength. This have suggested us that the dominant layer acts as a behaviour’s polarizer of the nodes in the other layers. We have redesigned the study of evolution considering the homophily as a shaping factor. In particular, we have studied its crucial role in breeding connections and rules interactions within a population, and then influence the strategies of players in multiplex. After having included these concepts of mutiplexity, communicability and homophily in our model, we have investigated the evolutionary dynamics both at macroscopic and microscopic scales. From one hand, the macroscopic evolution have highlighted the crucial role of homophily in solving the social dilemma, moving the population from the pure rational strategy (defection) towards the most profitable strategy with the highest evolutionary fitness (cooperation). From the other hand, the microscopic evolution has pointed out the impact of homophily on the formation of cooperative groups in the network and on groups’ size. The results have shown as homophily significantly affects the formation of cooperative groups, both in speed and size. Then, the introduction of multiplexity and homophily not only is a more realistic representation of social systems but, as shown in this work, it has a key effect on the evolutionary dynamics of cooperation.
